# Optimizing the Deep Neural Networks by Layer-Wise Refined Pruning and the Acceleration on FPGA

**DOI:** 10.1155/2022/8039281

**Published:** 2022-06-01

**Authors:** Hengyi Li, Xuebin Yue, Zhichen Wang, Zhilei Chai, Wenwen Wang, Hiroyuki Tomiyama, Lin Meng

**Affiliations:** ^1^Department of Electronic and Computer Engineering, Ritsumeikan University, Kusatsu, Shiga, Japan; ^2^School of AI and Computer Science, Jiangnan University, Wuxi, China; ^3^Department of Computer Science, University of Georgia, Athens, GA, USA

## Abstract

To accelerate the practical applications of artificial intelligence, this paper proposes a high efficient layer-wise refined pruning method for deep neural networks at the software level and accelerates the inference process at the hardware level on a field-programmable gate array (FPGA). The refined pruning operation is based on the channel-wise importance indexes of each layer and the layer-wise input sparsity of convolutional layers. The method utilizes the characteristics of the native networks without introducing any extra workloads to the training phase. In addition, the operation is easy to be extended to various state-of-the-art deep neural networks. The effectiveness of the method is verified on ResNet architecture and VGG networks in terms of dataset CIFAR10, CIFAR100, and ImageNet100. Experimental results show that in terms of ResNet50 on CIFAR10 and ResNet101 on CIFAR100, more than 85% of parameters and Floating-Point Operations are pruned with only 0.35% and 0.40% accuracy loss, respectively. As for the VGG network, 87.05% of parameters and 75.78% of Floating-Point Operations are pruned with only 0.74% accuracy loss for VGG13BN on CIFAR10. Furthermore, we accelerate the networks at the hardware level on the FPGA platform by utilizing the tool Vitis AI. For two threads mode in FPGA, the throughput/fps of the pruned VGG13BN and ResNet101 achieves 151.99 fps and 124.31 fps, respectively, and the pruned networks achieve about 4.3× and 1.8× speed up for VGG13BN and ResNet101, respectively, compared with the original networks on FPGA.

## 1. Introduction

Convolutional neural networks (CNNs) have brought significant revolutionary progress in almost all sciences with the availability of massive data and high-performance hardware since the milestone architecture AlexNet proposed in 2012 [1]. Deep neural networks (DNNs) have been applied for multiple tasks such as target classification, detection, and recognition. Various fields including computer vision, natural language processing [2], cultural heritage reorganization and protection [3], environment monitoring [4], Internet of Things [5], and service ecosystems [6], have been made the scientific breakthrough with the support of the technique. However, as DNNs achieve great performance improvement such as high accuracy with the deeper and larger size, problems follow (the codes related to the experiments of the study are available at: https://github.com/lihengyi-ai/2022CIaN).

The deeper architecture and larger size of DNNs take overwhelming computing resources with a large amount of redundancy [7]. The high intensity of computation and memory access leads to a heavy burden for deep neural networks, which is a huge obstacle that blocks the high-efficiency applications of artificial intelligence inference, especially for those resource-constrained hardware platforms. Then, it has been greatly important to compress and accelerate the DNNs from both the software level and hardware level, especially for those hardware resources and energy-limited terminal devices. Various studies have been made to optimize DNNs and have achieved significant progress. The solutions of software level can be briefly classified into four categories according to their properties [8]: pruning and quantization which reduce the computations to accelerate the network [9–11], low-rank factorization which utilizes the matrix/tensor decomposition to estimate the most informative parameters [12–14], transferred/compact convolutional filters which save parameters by designing the special structure for convolutional filters [15], and knowledge distillation [16] which trains a student network by utilizing the distilled knowledge from a large teacher model.

In terms of pruning technique, it is quite efficient for it significantly simplifies the network architecture by removing the redundant structures. At the same time, pruning operation also alleviates the overfitting effects of DNNs to a certain extent, as the previous study by Wan et al. [17] shows.

Research on pruning techniques mainly focuses on the training phase. The training-based methods utilize various algorithms such as L1 regularization to impose sparse constraints over specific parameters during the training process; thus, the unimportant architectures are identified by the sparse parameters and then be pruned[18–21]. However, the methods are costly and not very efficient for taking much more resources and time. In addition, many methods are constrained by various conditions, such as the parameters are not always sparse enough through L1 regularization. In this paper, based on the channel-wise importance analysis by utilizing the native characteristics of batch normalization (BN) layers and the convolutional layer-wise input sparsity, we propose a high-efficiency layer-wise refined pruning method at the software level.

As for the hardware-level technique, it complements the software-level solutions to accelerate the DNNs. The hardware platforms for DNNs include GPU, CPU, field-programmable gate array (FPGA), and application-specified integrated circuit (ASIC). The GPU and CPU are only software reprogrammable processors, which are only suitable for software-level optimization for DNNs. For the reason that ASIC is designed and manufactured for one specific function/algorithm and is not allowed to be modified once it is produced, it may be slightly deficient for the rapid update development of DNNs at present. At the same time, in terms of power consumption, which is a key factor for DNNs applications, Qasaimeh et al. [22] prove that FPGAs outperform GPUs and CPUs with 1.2–22.3× energy/frame reduction in terms of vision pipelines. In general, FPGA offers a promising solution for its characteristics of high flexibility, the nature of efficient parallel computing, high speed, power efficiency, and so on. The hardware platform makes full use of both hardware-level and software-level optimizing solutions for DNNs. In this paper, the original and the pruned network is further accelerated on the FPGA by utilizing the Xilinx Vitis AI. A comprehensive layer-wise performance analysis is also made in terms of the inference process on FPGA. The analysis found the possible bottlenecks that restrict the speed of the networks which indicates the points for improvement.

The application of DNNs contains the software as well as hardware. Previous research simply focuses on either the software-level or the hardware-level acceleration. This paper provides a “one-shot” solution that combines both. The main contributions of this article are as follows:

- Proposing a high-efficiency layer-wise refined pruning technique based on the channel-wise importance indexes according to the native characteristics of the parameters *γ* of BN layers and the layer-wise input sparsity of convolutional layers, without extra workloads for the training phase. Furthermore, the method is easy to be extended to all other neural networks that adopt BN operations.
- Implementing the pruned networks on the FPGA by utilizing the Vitis AI and making a comprehensive analysis of the performance of the DNNs on FPGA. Providing a thorough understanding and references of the DNNs on the FPGA platforms and indicating the research directions in the future study.

The rest of the paper is organized as follows.  Section 2 introduces the related work and the preliminary of the study.  Section 3 details the principle and the scheme of the layer-wise refined pruning method.  Section 4 presents the experimental results of the proposed pruning technique.  Section 5 accelerates the networks on FPGA and makes a comprehensive analysis of the neural networks on FPGA by the Vitis AI. At last, Section 6 concludes the paper.

## 2. Related Work

Although pruning is quite efficient to compress DNNs, the operation is also challenging. It is quite complicated to identify the redundant parts of the networks that can be pruned, and the pruning operation also makes changes for the adjacent layers which should also be considered. There has been much research on the technique and have made many achievements:

Liu et al. [23] proposed the network slimming technique to compress CNNs by imposing L1 regularization on the scaling factors in batch normalization layers during the training process. The method leads to the sparse distribution of the parameters. Thus, the redundant channels are identified and can be pruned to optimize the network. The method greatly decreases the parameters and memory consumption of the models and reduces the computational cost of state-of-the-art networks up to 20x compared with the original model, while with no accuracy loss. However, the L1 regularization does not always work for all such as the optimizer SGD hardly generates the sparse scaling factors.

He et al. [24] proposed a channel pruning method based on an iterative two-step algorithm. The approach takes the LASSO regression to identify the redundant channels of each layer with the least square reconstruction. The algorithm is applicable for multiple modern DNNs architectures, including VGGNet, ResNet, and Xception, and the experimental results show the effectiveness of the method: compared with the original network, the pruned VGG-16 achieves a 5× speedup with only 0.7% accuracy loss, and the pruned Xception achieves a 2× speedup with only 1.0 % accuracy loss.

Lym et al. [25] proposed the PruneTrain method which optimized the training from scratch of neural networks while pruning them for accelerating the inference process. The technique achieved a reduction in the computation cost of training modern networks by no less than half. Yang et al. [26] proposed the automatic neural network compression method to jointly prune and quantize DNNs. The proposal compressed the weights data of ResNet50 to be 836× smaller with no accuracy loss on CIFAR10.

Above are the related software-level pruning techniques for DNNs. As for FPGA, hardware description (HDL), e.g., Verilog and VHDL, has predominated the design since the beginning of FPGA, which is for register transfer level and logic synthesis into the target technology. However, HDL demands solid specialized knowledge at a low level for hardware and advanced hardware expertise. There have been some HDL-based accelerators for DNN: Yijin et al. [27] designed the Crane accelerator. The hardware solution utilizes all kinds of sparsity including the irregular sparsity in CNNs and improves the CNNs performance by a 27–88% speedup and energy saving of 16–48% compared with the state-of-the-art prior approaches.

However, the difficulty and time-consuming characteristics of HDL have greatly stunted the FPGA-based design. Under this circumstance, high-level synthesis (HLS) emerged as an effective solution, which allows high-level language, such as C and C++, to become hardwired specifications in HDL. HLS-based accelerator design has also made much progress for DNNs: accelerator PipeCNN has achieved up to a 37× performance improvement for CNN-based applications [28]; the FPGA-accelerated binarized networks outperform the corresponding GPU-accelerated counterparts by achieving a \gt 7-fold decrease in power consumption while 2.86× faster for inference tasks [28]. Sarg et al. [29] proposed a C++ HLS implementation for FPGA-based accelerators and achieved up to a 339× inference speedup for ResNet50.

Compared with the HLS tools, the Vitis AI, a development stack for AI inference on Xilinx hardware platforms, simplifies and improves the efficiency of AI acceleration on Xilinx FPGA furthermore. There have been many studies for accelerating DNNs by utilizing the Vitis AI. Kedia et al. [30] proposed a design space exploration strategy, which achieved a 28× reduction in the number of design points to be simulated and a 23× speedup in pruning time for FPGA-based DNNs systems. Wang and Gu [31] implemented the YOLOv3 network on Xilinx FPGA by using the Vitis AI, and the hardware accelerator achieves a lower energy consumption and higher throughput compared with the GPU implementation.

This paper focuses on the study of channel pruning from the software level and accelerates the optimized networks on the FPGA platform with the support of the Vitis AI development tool from the hardware level.

## 3. Layer-Wise Refined Pruning

As is presented above that DNNs are heavily overparameterized and the pruning technique is a highly efficient solution to compress the redundancies. In this paper, a layer-wise refined pruning to optimize the overparameterized DNNs is proposed, the principle.

The key to pruning the network is how to identify the redundant parts that can be pruned. Although the sparsity indicates the massive redundancy of DNNs that have a large number of meaningless computations, it does not point out the redundant structures concerning these meaningless operations. In this paper, the redundant units of a neural network are identified based on the following two factors:

- Layer-wise sparsity: first, we analyze the input sparsity of each convolutional layer to obtain a refined, quantitative, layer-wise redundancy of the network.
- Channel-wise importance indexes: second, we obtain the channel-wise importance indexes of each layer based on the native characteristics of BN layers.

Then, the redundant channels of each layer, which can be pruned, are identified based on the convolutional layer-wise input sparsity and the channel-wise importance indexes according to BN layers. First, Subsections 3.1 and 3.2 detail the principle of the proposal. And then, Subsection 3.3 introduces the scheme of the method.

The study takes ResNet [32] and VGGBN [33] architectures to verify its proposal for the following points. First, ResNet addresses the degradation as well as the saturation or even degrading rapidly of the gradient of DNNs. It makes it possible to train deep-depth neural networks that have up to hundreds or even thousands of layers and still achieve compelling performance. As a result, it is arguably one of the most breakthrough architectures in computer vision concerning deep depth artificial learning and is widely referenced by follow-up studies since its proposal [34, 35]. Second, VGG architecture is a milestone for DNNs for its powerful representational ability in feature extraction is incomparable.

### 3.1. Layer-Wise Dynamic Sparsity

**Figure 1:**
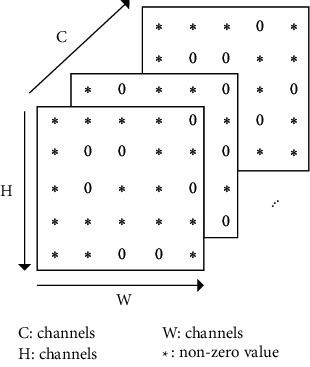
The input feature maps of a certain convolutional layer.

Sparsity refers to the zero elements of a matrix that are meaningless to the calculation. The sparsity in DNNs can be simply classified into two categories: dynamic sparsity and static sparsity, which refer to the sparsity of input/output feature maps that varies with different inputs, and parameter sparsity which is determined after training, respectively. In this paper, the proposal focuses on dynamic sparsity. The feature-map sparsity is largely attributed to the Rectified Linear Unit (ReLU) activation function [36]. As the input feature maps of a certain layer can be shown in Figure 1, the dynamic sparsity can be expressed as follows:

(1) $$\begin{array}{c} DS\left(n\right)=\frac{\sum_{i=0}^{C}\sum_{j=0}^{H}\sum_{k=0}^{W}f\left(O\left(i,j,k\right)==0\right)}{C\times H\times W}, \end{array}$$

where *DS*(*n*) denotes the input sparsity of the *n*^*th*^ convolutional layer; *O*(*i*, *j*, *k*) denotes the element of the *i*^*th*^ channel, the *j*^*th*^ row, and the *k*^*th*^ column of the feature maps; the function *f*(*∗*) defines the judgment operation of *O*(*i*, *j*, *k*) and equals 1 in case that *O*(*i*, *j*, *k*) is 0; otherwise, it equals 0.

**Figure 2:**
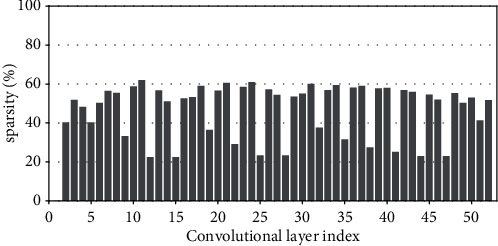
Sparsity of ResNet50 on CIFAR10.

**Figure 3:**
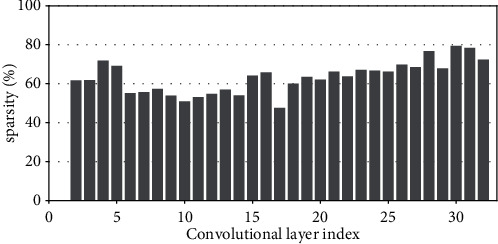
Sparsity of ResNet50 on CIFAR100.

**Figure 4:**
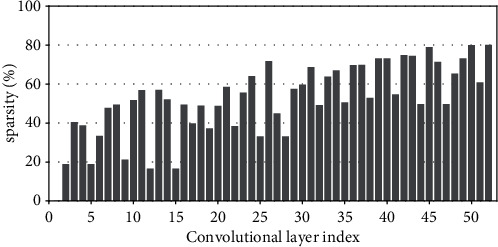
Sparsity of ResNet50 on ImageNet100.

**Figure 5:**
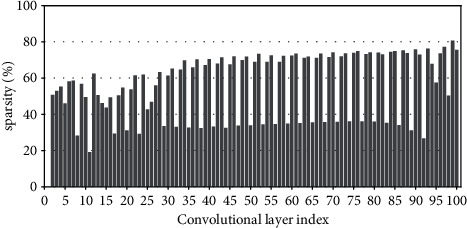
Sparsity of ResNet101 on CIFAR100.

**Figure 6:**
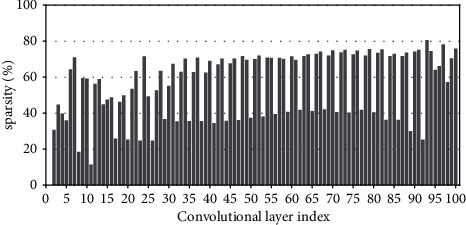
Sparsity of ResNet101 on ImageNet100.

**Figure 7:**
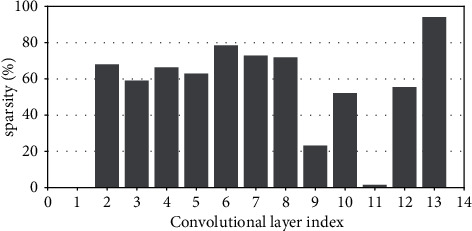
Sparsity of VGG13BN on CIFAR10.

**Figure 8:**
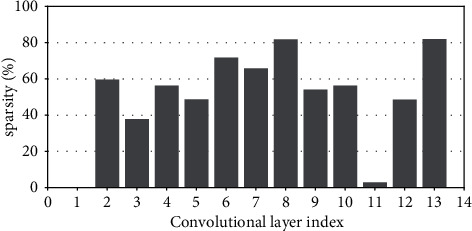
Sparsity of VGG13BN on CIFAR100.

**Figure 9:**
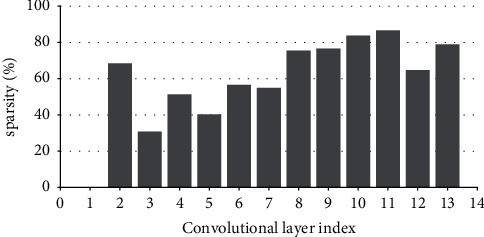
Sparsity of VGG13BN on ImageNet100.

Research has shown that the input feature-map sparsity of convolutional layers is no less than 50 % on average for the state-of-the-art DNN models on dataset ImageNet, including VGGNets, InceptionNets, ResNets, and DenseNets[7]. Even for the dataset with a small figure size of 32 × 32, such as CIFAR10, the dynamic sparsity is still very high. For example, the input sparsity of convolutional layers is 46.79% on average for ResNet50 on CIFAR10. For clarity, Figures 2–9 show the input feature-map sparsity concerning the experiments of the networks and datasets in the paper, including ResNet50, ResNet101, and VGG13BN on dataset CIFAR10 and ImageNet100, and the results are averaged from 100 inputs. For the reason that the computations concerning the sparsity are all meaningless, a large number of redundant computations can be pruned to optimize the network.

Although the feature-map sparsity reflects the redundancy of DNNs, it varies with different inputs. Utilizing the activation sparsity to prune the network has to take the difference into account. We analyze the Coefficient of Variation (CV) of convolutional layers' input sparsity, which proves the feasibility and reliability to take randomly one input to get the layer-wise sparsity of a network for pruning. CV is defined as the ratio of the standard deviation to the mean to show the extent of variability concerning the mean of the data as equation (2) shows, which shows the extent of variability about the mean of the data.

(2) $$\begin{array}{c} CV_{i}=\frac{\sigma_{i}}{\mu_{i}}, \end{array}$$

where *CV*_*i*_ denotes the input sparsity CV of the *n*^*th*^ convolutional layer, *σ*_*i*_ denotes the input sparsity standard deviation and *μ*_*i*_ denotes the input sparsity mean of the *n*^*th*^ convolutional layer, respectively.

**Figure 10:**
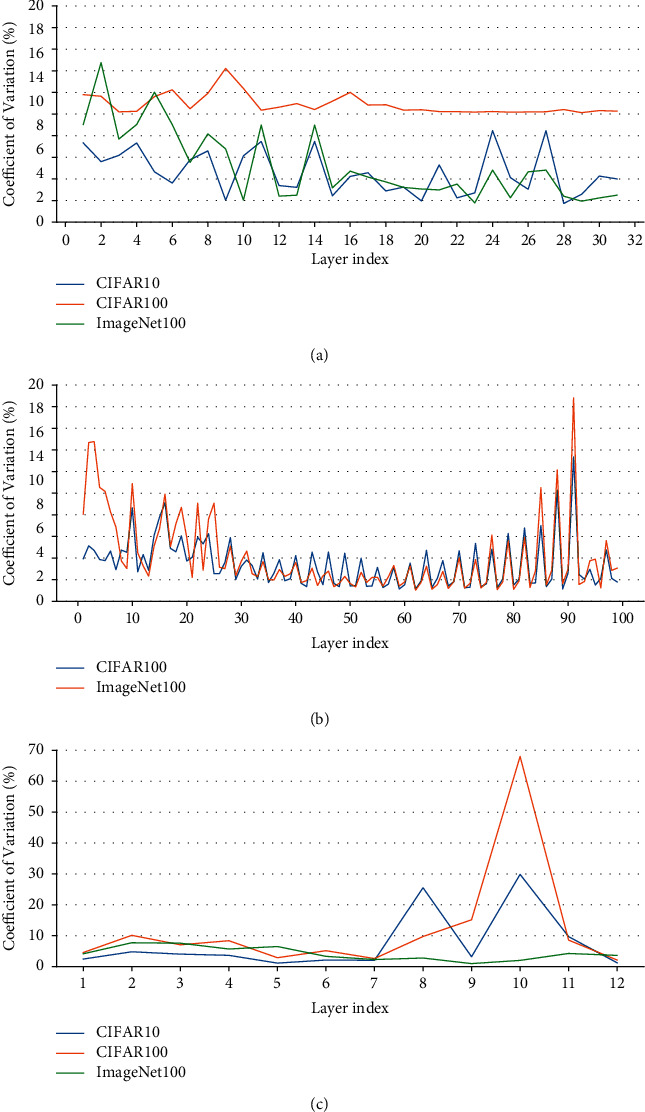
CV of input sparsity. (a) ResNet50. (b) ResNet101. (c) VGG13BN.

Figure 10(a) shows the CV of the convolutional layers' input sparsity of ResNet50 except for the first convolutional layer which has the original input data, and the values of the CV are no more than 15 % for all.  Figure 10(b) shows the convolutional layer-wise input sparsity CV of ResNet101 except for the first layer. The values of the CV which are higher than 9% correspond to the three convolutional layers of the first residual block and the first convolutional layer of the other residual blocks of the ResNet101, which are not the pruning objects in the paper.  Figure 10(c) shows the convolutional layer-wise input sparsity CV of VGG13BN except for the first layer. Although the CV of both the 10^*th*^ convolutional layers' input sparsity of VGG13BN on CIFAR10 and CIFAR100 is much higher than 15%, it needs to be noted that the corresponding input sparsities are much smaller, which are both less than 5% as Figures 7 and 8 show. As for the CV of the 8^*th*^ convolutional layers' input sparsity of VGG13BN on CIFAR10 which is also higher than 15%, the input sparsity is also much less than others with the value of about only 22%.

Based on the statistics or mathematical analysis, the CV with a value that no more than 15 % indicates that the data varies within a small fluctuation range. The results shown in Figure 10 prove that the refined pruning method can just adopt the feature-map sparsity of a random input while ignoring the little difference caused by different inputs.

### 3.2. Channel-Wise Important Indexes

**Figure 11:**
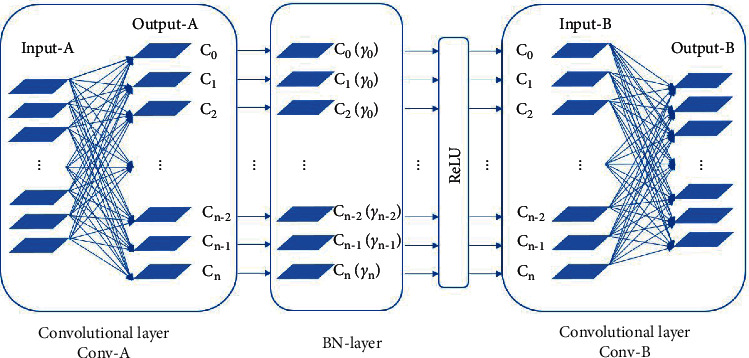
The typical substructure of DNNs.

In this paper, the channel-wise importance indexes are obtained based on the parameters *γ* of BN layers. And the principle is detailed in this section. For clarity, Figure 11 shows the typical substructure of DNNs with the Batch normalization operation and the two adjacent convolutional layers. Assume that the input channels of convolutional layer Conv-A are less than the output channels *n* and the output channels of convolutional layer Conv-B are less than the input channels *n*. And the output channels of Conv-A, the channels of the BN layer, and the input channels of Conv-B are all *n* which is determined by the principle of DNNs.

Batch normalization was proposed to mitigate the Internal Covariate Shift caused by the change of parameters in the network during the training process by Sergey Loffe and Christia Szegedy in 2015 [37]. The technique has been adopted by most of the state-of-the-art representative DNNs architectures, including VGGBN [32], InceptionNet [37–39], ResNet [32], DenseNet [40], MobileNet [41], ShuffleNet [42, 43], and MnasNet [44]. The mathematical function of batch normalization is defined as follows:

(3) $$\begin{array}{c} \hat{\text{activation}}=\frac{{\text{activation}}_{\text{in}}-\mu_{B}}{\sqrt{\sigma_{B}^{2}+\epsilon }},\\ Y=\gamma \times \hat{\text{activation}}+\beta , \end{array}$$

where $\hat{\text{activation}}$ denotes normalized values of activation, and *Y* denotes linear transformations of $\hat{\text{activation}}$, which is the output of the *BN* layer. Parameters *μ* and *σ* denote the mean and the standard deviation of input activation, respectively. The two parameters are calculated per dimension over the minibatches. As a constant, *ϵ* is for numerical stability. *B* denotes the minibatch, i.e., equals 20 in this paper. The parameters that need to be trained for BN layers are *γ* and *β*: *γ* is also named weights, and *β* is named bias in PyTorch. The two factors are set to be 1 and 0 by default before training. By normalizing the activation with recentering and rescaling operation, the batch normalization technique speeds up the convergence process and improves significantly the generalization ability of DNNs.

In terms of the parameters *γ*, for a certain BN layer, it needs to be noted that each channel has a corresponding *γ*. For clarity, we define *c*_*j*_(*γ*_*j*_) as follows.

**Definition 1 .**

*c*_*j*_(*γ*_*j*_):

For a certain BN layer, it consists of *n* channels *c* ∈ *C* : [*c*_0_, *c*_1_, *c*_2_,…, *c*_*n*_]; there are also *n* elements of *γ* ∈ Γ : [*γ*_0_, *γ*_1_, *γ*_2_,…, *γ*_*n*_], where *C* and Γ are a consistent one-to-one match. Then, *c*_*j*_(*γ*_*j*_) denotes the *j*^*th*^ channel and the corresponding *j*^*th*^*γ* of a certain BN layer.

In the study, we make a depth analysis of the characteristic of parameters *γ* and find an important property. And it is exactly the *γ* factor that plays a crucial role in our proposal.

We map the density of the parameters *γ* in terms of VGG13BN and ResNet101 which are trained on the dataset of ImageNet100. The density map is a representation of the distribution of the data with axis-*X* denoting the values of the data, axis-*Y* denoting the density of the value, and the interval area indicating the distribution frequency of the data located in the interval.

**Figure 12:**
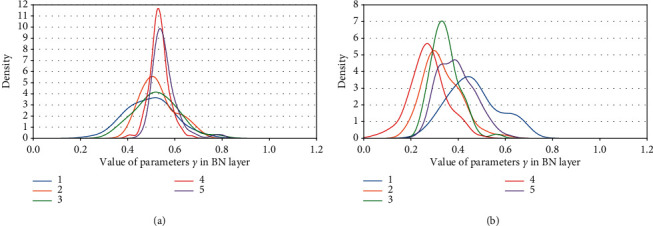
Kernel density of parameters *γ*. (a) ResNet50 on CIFAR10. (b) ResNet50 on CIFAR100.

**Figure 13:**
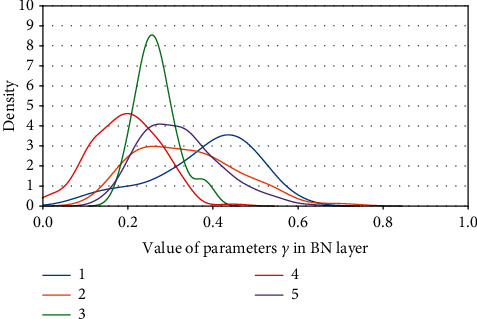
Kernel density of parameters *γ*: ResNet50 on ImageNet100.

**Figure 14:**
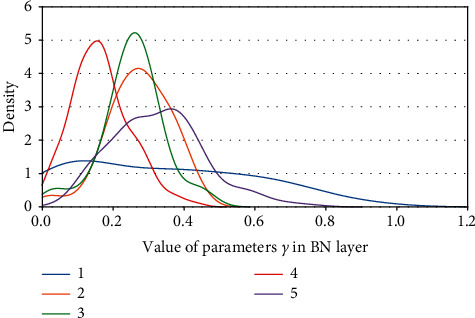
Kernel density of parameters *γ* s: ResNet101 on CIFAR100.

**Figure 15:**
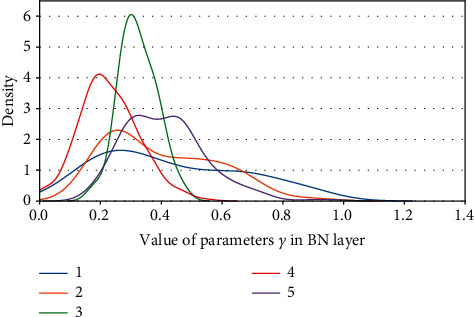
Kernel density of parameters *γ*: ResNet101 on ImageNet100.

**Figure 16:**
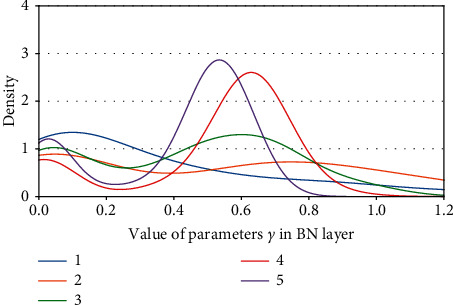
Kernel density of parameters *γ*: VGG13BN on CIFAR10.

**Figure 17:**
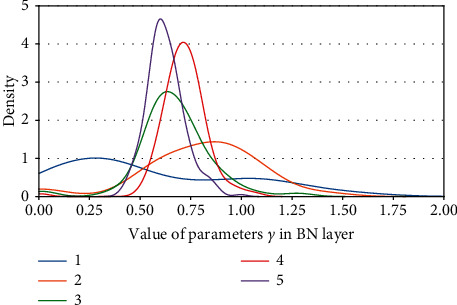
Kernel density of parameters *γ*: VGG13BN on CIFAR100.

**Figure 18:**
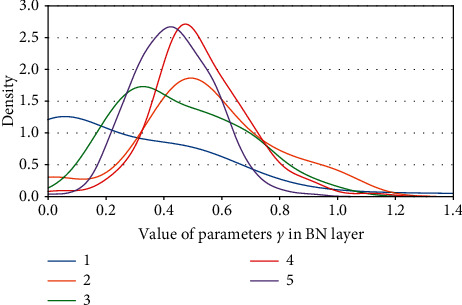
Kernel density of parameters *γ*: VGG13BN on ImageNet100.

Figures 12(a) and 12(b) show the distribution of parameters *γ* of ResNet50 on CIFAR10 and CIFAR100 with density map, respectively. For clarity, the figures just represent data of the first five BN layers, and the other parts share a similar distribution with the first five BN layers except for the distribution regions. Furthermore, Figures 13–18 in provide more detailed distribution of parameters *γ* about networks ResNet50, ResNet101, and VGG13BN on CIFAR10, CIFAR100, and ImageNet100. As can be seen, the data are in approximately normal distribution and the distribution region varies with different layers, except for the 5th BN layer for VGG13BN.

Although the network slimming technique [23] also utilizes the scalar factors of BN layers, there is a fundamental difference in our proposal. The network slimming technique is based on the theory utilizing L1 regularization on the *γ* factor of BN layers to sparse the parameters during training. However, the method does not always work and the weights of the BN layers do not always represent the sparse distribution. For example, in the cases that the optimizer adopts SGD and especially during the process of the multipass learning scheme. And the approximately normal distribution is the most scenic. At the same time, the method requires more training epochs for the network to converge.

**Figure 19:**
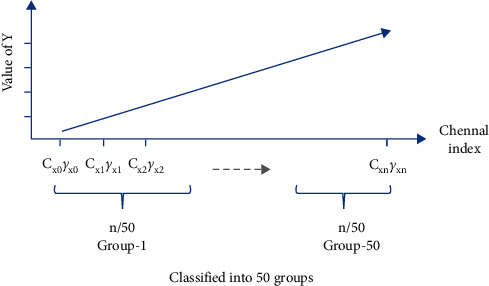
50-groupwise pruning experiment.

In the study, we make a 50-group-wise-pruning experiment with group-wise pruning to show that the values of parameters *γ* indicate the importance of the corresponding channels to a great extent. As Figure 11 shows, the channels of the BN layer, as well as the output channels of the convolutional layer Conv-A and the input channels of convolutional layer Conv-B, are one-to-one match consistent. Then, they can be seen as the basic pruning unit, and the pruning operation for them is centered on the BN layer. The 50-group-wise-pruning experiment is detailed as follows:

- First, the channels of the three layers are sorted in ascending order based on the values of the parameters *γ* of the BN layer respectively. As Figure 19 shows, *γ*_*x*(*m* − 1)_ ≤ *γ*_*xm*_, *c*_*xm*_(*γ*_*xm*_) denotes the certain *m*^*th*^ channel and *γ* in the sorted order, and *n* denotes the number of channels of the BN layer. It needs to be noted that the arrow in Figure 19 just indicates the increase of values of parameters *γ* that *γ*_*x*(*m* − 1)_ ≤ *γ*_*xm*_, while not the actual change of the values. Due to the consistency of the channels of the three layers, the arrangement order of the channels for the three layers is also consistent.
- Second, all of the channels of each layer of the network based on the substructure shown in Figure 11 are sorted in the same method.
- Third, as Figure 19 shows, the channels of each layer are classified into 50 even parts, respectively, based on the arranged order, namely, based on the values of corresponding parameters *γ* from low to high. The number of channels of each group equals ⌈*n*/50⌉.
- Finally, the 50 even parts of channels are pruned in order from low to high, respectively. And the accuracy of the pruned model is tested on the validation dataset, respectively.

**Figure 20:**
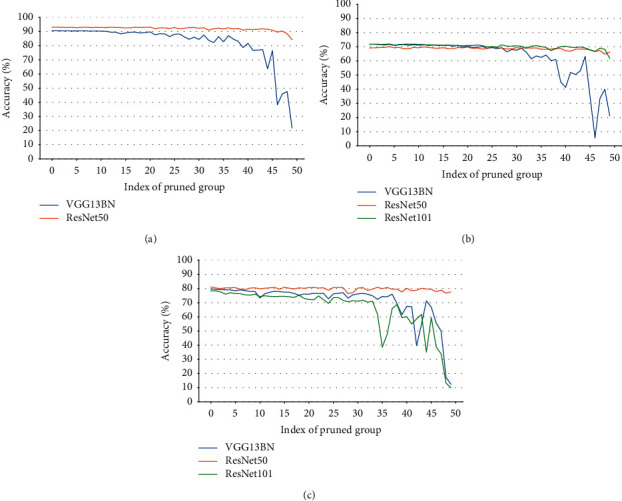
Accuracy of groupwise pruning. (a) Networks on CIFAR10. (b) Networks on CIFAR100. (c) Networks on ImageNet100. Group 1 has the lowest parameters *γ*; Group 50 has the highest parameters *γ*.

Considering the residual architecture of ResNet, the input channels and the output channels of the residual block remain unchanged.  Figure 20 shows the results of the 50-group-wise-pruning experiment about the three models tested on three kinds of datasets, with axis-*X* denoting the group index and axis-*Y* denoting the validation accuracy. For the reason that ResNet101 is too powerful for CIFAR10, we just test ResNet50 and VGG13BN on CIFAR10. Conclusions can be drawn as follows, which provides the foundation for channel pruning to judge which channels are less important or redundant that can be pruned:

- The results concerning ResNet50 on CIFAR10 and CIFAR100 and ImageNet100 and ResNet101 on CIFAR100 show that the validation accuracy decreases slightly as the group of channels with increasing values of parameters *γ* is pruned especially for the first twenty groups of channels. Besides sparsity, the phenomenon also strongly demonstrates that there are massive redundancies in DNNs introduced in Section 2.
- The results concerning VGG13BN on CIFAR10 and CIFAR100 and ResNet101 and VGG13BN on ImageNet100 show that the validation accuracy decreases slightly for the groups of channels in about the first 30 groups are pruned. However, the accuracy decreases greatly when the group of channels in the last 20 groups is pruned, especially for the last 10 groups of channels. To be exact, it is the last 30% channels that weigh much for VGG13BN and the last 35% channels that weigh much for ResNet101. Especially for the last group of the 50 even parts, the validation accuracies of the two networks decrease by more than 80%. The phenomenon indicates that the parameters *γ* represent the importance of the channels of each layer to a great extent. That is, the channels of each layer in the network with small values of parameters *γ* are less important and even redundant for the DNNs architecture, and the channels with larger values of parameters *γ* are more important for the network.

As a result, the parameters *γ* can be used as the channel-wise importance indicators of each layer to identify the redundant channels for pruning the network.

### 3.3. Layer-Wise Refined Pruning Method

**Figure 21:**
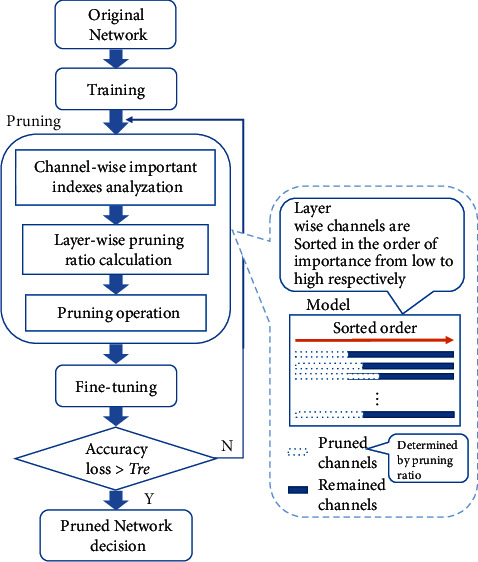
Layer-wise refined pruning flow chart.

Based on the input feature-map sparsity of convolutional layers and the channel-wise importance indicators of each layer, the high-efficiency layer-wise refined pruning approach is proposed.  Figure 21 shows the overall flow chart to optimize a deep neural network based on the layer-wise refined pruning method. Given the original network *M*_*o*_, first, the network is trained on the target dataset from scratch. Second, the trained network is compressed by the proposed layer-wise refined pruning approach. Third, after the pruning operation, the pruned network is fine-tuned to compensate for the accuracy loss; i.e., the pruned network is retrained. The pruning operation, as well as the fine-tuning operation, is iterated multiple times to get a higher pruning ratio until the accuracy of the validation dataset decreases by more than 1.5%. And finally, a highly optimized network is obtained.

The layer-wise refined pruning method is detailed as follows, and it takes four steps. For clarity, Figure 11 shows the typical structure of DNNs with a BN layer, which is also taken as the basic pruning unit and used to illustrate in detail the proposal.

**Figure 22:**
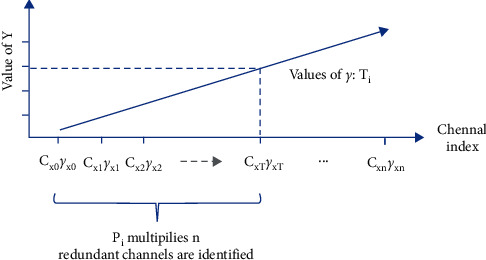
Redundant channels identification of a specified BN layer.

First, the channels of each layer are sorted in the order of importance from low to high, respectively: As has been proved that although the parameters *γ* of batch normalization layers are in a similar normal distribution, the values reflect the importance of the correlated channels to a great extent, Then, the channels of the BN layers are sorted, respectively, in the order of their importance according to the parameters *γ*, as is shown in Figure 22. Likewise, the adjacent convolutional layers' channels, i.e., the output channels output-A of Conv-A and the input channels input-B of Conv-B, share the consistent characteristic: the channels are a consistent one-to-one match between channels of output-A and input-B. Then, they are sorted in the same order as that of the BN layers. Thus, the channels are sorted in the same order.

Second, the layer-wise pruning ratios are calculated. As the input-B feature-map sparsity reflects the level of redundancy, thus the pruning ratio, i.e., the proportion of redundant channels concerning the output of Conv-A, BN layer, and input of Conv-B that can be pruned, is closely related to the sparsity and can be determined according to the feature-map sparsity. The pruning ratio P[ *P*_0_, *P*_1_, *P*_2_ ,…, *P*_*N*_ ] is defined as follows:

(4) $$\begin{array}{c} P_{i}=\left\{\begin{array}{c} pct_{i}\text{if }pct_{i}<=\alpha ,\\ pct_{i}\times \eta \text{ if }pct_{i}>\alpha \text{ and }\eta \in \left(0,1\right], \end{array}\right. \end{array}$$

where *P*_*i*_ defines the pruning ratio of the *i*^*th*^ BN layer, the output of Conv-A, and the input of Conv-B. *pct*_*i*_ denotes the input sparsity of the convolutional layer that follows the *i*^*th*^ BN layer. To avoid a much higher pruning ratio, the pruning ratio would be *pct*_*i*_ × *η* in case the input sparsity is higher than *α*. It is detailed with the experimental data in Section 4.2 about how to make the values of the two parameters.

Third, the redundant channels that can be pruned are identified. As Figure 22 shows, in terms of a certain BN layer, based on the pruning ratio and the sorted channels in the order according to *γ*, the thresholds, T[ *T*_0_, *T*_1_, *T*_2_ ,…, *T*_*N*_ ] where *T*_*i*_ is defined as the pruning threshold with regard to the parameters *γ* of the *i*^*th*^ BN layer, are obtained by multiplying the pruning ratio with the number of channels N. Based on the set of scalars within range of the pruning ratio that is smaller than the threshold, the correlated channels of the BN layer are identified as redundant. And the convolutional channels of output-A and input-B in accordance with these identified channels are all screened out as redundant. Then, all of the redundant channels of the network are identified based on the principle detailed above.

Finally, the pruning operation is executed. The pruning objects include the redundant channels and the corresponding parameters for both convolutional layers and the batch normalization layers. Although the pruned objects are greatly redundant for the network, they also have a certain value, which results in the accuracy decrease when they are removed from the network. For this reason, the pruned network is fine-tuned by retraining the pruned model to compensate for the accuracy loss. And to achieve a high pruning ratio, the pruning operation is iterated multiple times until the accuracy decreases more than a threshold *Tre* and cannot be compensated by fine-tuning. In this paper, the *Tre* is set to 1.5%. And the fine-tuning scheme also refers to knowledge distillation to a certain extent. Algorithm 1 illustrates the process of the pruning operation as well as the fine-tuning process. Eventually, a highly optimized model is obtained.

## 4. Refined Pruning Experiments

This section represents the experimental results of the proposed layer-wise refined pruning method.

### 4.1. Preliminary

The deep learning framework applied is the PyTorch of version 1.9.0 + cu102, one of the representative open-source frameworks for machine learning. The hardware platform is Intel(R) Core(TM) i9-10900 CPU @ 2.80 GHz, 4 × 16 GB DDR4 main memory, and GeForce GTX 3080 Ti with CUDA version 11.4. The operating system is Ubuntu 20.04.

CIFAR10 and CIFAR100 [45] and ImageNet [46] are adopted as Benchmark datasets to make the experiments. Due to the limitations of experimental resources, the entire ImageNet database with 1000 object classes and about 1.28 million training images is too large for the hardware platform. Then, 100 classifies of ImageNet Dataset are randomly selected as ImageNet100 which consists of 129026 items in this study. And the ImageNet100 is split into three parts in the proportion of 16 : 4:5, i.e., the training set, validation set, and test set. The training data are preprocessed with a random crop, random horizontal flip, random rotation, and normalization of mean and standard deviation for the three datasets. The accuracy calculated in the paper is tested on the validation dataset.

For the ResNet and VGGBN architecture, ResNet50, ResNet101, and VGG13BN are taken to verify the effectiveness of the pruning method on CIFAR10, CIFAR100, and ImageNet100, respectively, which cover the two neural network architectures as well as a wide range of convolutional layers in number from thirteen to more than one hundred. In the experiments, the three models are trained on the three datasets from scratch. In terms of the hyperparameters for the training process, the optimizer is the momentum-accelerated stochastic gradient descent (SGD)[47], with the momentum of 0.9, weight decay of 0.0001, Nesterov. The training epochs are set to 100 and 50 for the initial training and fine-tuning process, respectively, with the batch size both set to 20. In terms of the learning rate, we take the cosine annealing schedule, which is adopted in this paper defined as follows [48].

(5) $$\begin{array}{c} lr\left(n\right)=lr_{\min}+\frac{1}{2}\left(lr_{\max}-lr_{\min}\right)\left(1+\cos\left(\pi \right)\right), \end{array}$$

where lr (*n*) denotes the learning rate of the *n*^*th*^ epoch and *lr*_min_ and *lr*_max_ denote the initial and the minimum learning rate which are set to 0.01 and 1*e* − 5 in this paper. As for the refining process, except for the epochs being set to 50, all of the other parameters are the same. Since the experiment adopts the same hyperparameters for the training process, some networks may not get the best result in the validation accuracy.

### 4.2. ResNet

**Figure 23:**
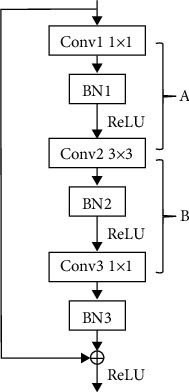
A “bottleneck” building block for ResNet101.

As for ResNet50 and ResNet101, the residual block, as Figure 23 shows, is treated as the basic pruning unit. Considering the consistency of the residual architecture, the input channels of the first convolutional layer Conv1 and the output channels of the last convolutional layer Conv3 within a residual block remain unchanged. The pruning operation is divided into two groups within a block:

- The pruned channels of Conv1-output, BN1-input/output, and Conv2-input are all identified by BN1
- The pruned channels of Conv2-output, BN1-input/output, and Conv2-input are all identified by BN2

And then, the redundant channels, as well as the correlated parameters, are pruned from the network.

In terms of the pruning ratio shown in equation (4), we make comparisons between ResNet50 on CIFAR10 and ResNet101 on CIFAR100 by taking different pruning ratio schemes:

- Scheme 1: Tables 1 and 2 show the results of the parameters *β* and *γ* which are both set to 0.5; it takes 8 pruning iterations to get a better-pruned network for ResNet50 on CIFAR10, with the parameters and FLOPs pruned by 86.18% and 88.64%, respectively. As for ResNet101 on CIFAR100, it takes 5 iterations to get a better-pruned network with parameters and FLOPs pruned by 87.12% and 88.65%, respectively. And the accuracy loss is 1.31% and 0.40%, respectively.
- Scheme 2: Tables 3 and 4 show the results of the parameters *β* and *γ* which are both set to 1; it takes only 3 pruning iterations to achieve a better-pruned network for ResNet50 on CIFAR10 with the parameters and FLOPs pruned by 84.27% and 86.34%, respectively. As for ResNet101 on CIFAR100, it takes 3 iterations to get a better-pruned network with parameters and FLOPs pruned by 87.45% and 88.19%, respectively. And the accuracy loss is 0.88% and 1.01%, respectively.

It is obvious that the pruning operation could be more refined to achieve a higher pruning ratio while taking more iterations for the two parameters *β* and *γ* adopting small values, but it takes less pruning iterations with less pruning ratio for the two parameters adopting high values. So the values for the two parameters need to be weighed in the operation between the pruning ratio and the pruning iterations. In general, it is appropriate for the two parameters to take no less than 0.5 considering the margins. The value of 0.5 is deduced from Figure 20, the accuracy of group-wise pruning experiments, which shows that about 60% of channels corresponding to the lower parameters *γ* are less important for the networks. And the value is also verified by the experimental results above.

Table 5 shows the results of related studies concerning ResNet50 on CIFAR10. For the reason that the baseline accuracy varies with training conditions, such as epochs and optimizers, besides providing baseline validation accuracy of various techniques, the percent change of validation accuracy is taken as the comparative index. The 6^*th*^ iteration results in Table 1 show the superior of our proposal to other methods: more than 83.97% of parameters and 86.30% of FLOPs are pruned with 0.4% validation accuracy improvement, as well as the best validation accuracy after pruning except for the technique MS-KD. As for other techniques, the validation accuracy decreases more or less except for AutoML for model compression with only a 0.12% improvement. Considering the baseline validation accuracy which is closely related to the training conditions, although the validation accuracy is 1.21% lower than that of MS-KD, the validation accuracy improvement by 0.40% of the 6^*th*^ iteration with our proposal still proves superior to MS-KD.

As for CIFAR10 and CIFAR100, they are similar to each other except that CIFAR10 has 10 classes containing 6000 images per class, while CIFAR100 has 100 classes containing 600 images each. For this reason, we extend the pruning experiments of ResNet50 and ResNet100 on ImageNet100 to verify the effectiveness of the proposal.  Table 6 shows the results of ResNet50 on ImageNet100. The validation accuracy only decreased by 0.85% when 78.49% of parameters and 78.24% of FLOPS are pruned in the 4^*th*^ pruning iteration with the accuracy decreased by 0.85%. And the accuracy decreased by 2.38% when 81.86% of parameters and 82.04% of FLOPS are pruned in the 5^*th*^ pruning iteration.  Table 7 shows the results of ResNet101 on ImageNet100. The pruning operation is iterated four times. And the validation accuracy only decreased by 0.70% when 84.22% of parameters and 84.36% of FLOPS are pruned in the 3^*r*  *d*^ pruning iteration. And the accuracy decreased by 1.71% when 87.29% of parameters and 87.71% of FLOPS are pruned in the 4^*th*^ pruning iteration.

In terms of ResNet50 and ResNet101 for which convolutional layers vary from fifty-two to more than one hundred, the proposal achieves a considerable pruning ratio for both parameters and FLOPS with no more than 1.5% accuracy loss. Besides, an important conclusion is drawn from the experimental results that the deeper network does not absolutely perform better: for ImageNet100, the 4^*th*^ iteration pruned network of ResNet50 performance is obviously superior to the 3^*r*  *d*^ iteration pruning network, with 7.44% less of parameters and 20.18% less of FLOPS while 1.30% higher of validation accuracy. The phenomenon also proves the effectiveness of the proposed method to obtain an optimized network for inference applications.

### 4.3. VGG13BN

In terms of VGG architecture, we take VGG13BN to verify the effectiveness of the proposal. For VGG13BN, all of the convolution layers, as well as the fully connected layers, are compressed. To utilize the refined pruning method for the whole architecture, the last three fully connected layers are implemented with convolution computing followed by the BN layer and ReLU, which has little effect on the performance of the model. The pruning ratio for each layer is up to the input sparsity of the layer with *α* and *η* both being set to 0.5.

Table 8 shows the results on CIFAR10. The pruning operation is iterated three times. And the validation accuracy only decreased by 0.74% when 87.05% parameters and 75.78% FLOPS are pruned for the 3rd pruning iteration. And the accuracy decreased by 3.21 % when 93.02 % parameters and 85.01 % FLOPS are pruned for the 4th pruning iteration.

Table 9 shows the results on CIFAR100. The pruning operation is iterated two times. The validation accuracy increased by 1.27% when 60.31% parameters and 33.77% FLOPS are pruned for the 2nd pruning iteration. And the accuracy decreased by 1.55% when 78.45% parameters and 62.62% FLOPS are pruned for the 4th pruning iteration.

Table 10 shows the results on ImageNet100. The pruning operation is iterated three times. The validation accuracy only decreased by 1.27% when 84.76% parameters and 79.47% Floating-Point Operations (FLOPS) are pruned. And the accuracy decreased by 5.33% when more than 90% parameters and FLOPS are pruned.

### 4.4. Results Analysis

In this section, we make a series of experiments to verify the effectiveness of the proposed refined pruning method. The proposal achieves a higher pruning ratio on both parameters and FLOPs with no more than 1.5% accuracy loss, and the optimized layer-wise pruning threshold also avoids the unbalanced pruning that almost all channels of some layers are pruned while little for others caused by the global threshold which [23] adopts. By adjusting the two pruning ratio parameters *α* and *η* in (4) to smaller, the pruning operation makes more parameters and FLOPs pruned with less accuracy loss while just taking more pruning iterations. So the values for the two parameters need to be weighed in the operation between the pruning ratio and the pruning iterations. However, although it takes more pruning iterations for the training process to get a higher pruning ratio by setting the parameters *α* and *η* to smaller, it is worthy for the final deployment and application of the trained models which actually takes less memory, fewer FLOPs, and less inference time.

In terms of the overall flow since training the original network from scratch to obtain the highly optimized model for inference applications, it is exactly the training and the fine-tuning process that dominate the time cost, which is closely related to the hardware, i.e., the performance of the GPUs. Hence, the time for training and fine-tuning is not listed as key parts. As for the proposed refined pruning operation, the time cost is negligible: for the first pruning iteration which takes the most time, ResNet50 on CIFAR takes the least time that is 2.76 ms; ResNet101 on ImageNet100 takes the most time that is 8.27 ms. And the data is averaged from 100 iterations.

At the same time, the phenomenon that all of the validation accuracies of the experiments increase more or less than those of the baseline for the first several iterations strengthens the argument that the pruning operation does also improve the overfitting of DNNs.

## 5. Acceleration and Analysis of DNNS on FPGA

In this section, we deploy both the original and the pruned VGG13BN and ResNet101 trained on ImageNet100 on the Xilinx FPGA platform of Zynq UltraScale + MPSoC ZCU102 Evaluation Kit. As for the pruned networks, the pruning iteration-2 network of VGG13BN and the iteration-3 network of ResNet101 are adopted.

**Figure 24:**
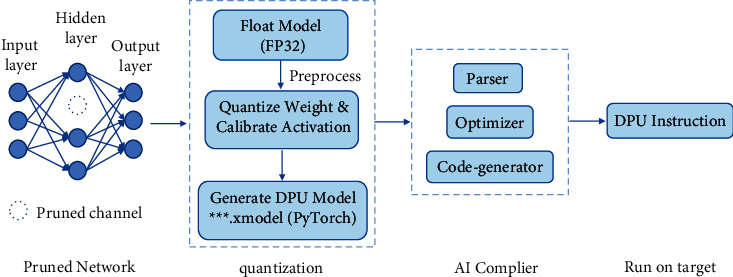
The Vitis AI flow.

The Vitis AI [52] offers an adaptable and real-time AI inference acceleration solution for Xilinx hardware platforms, which includes a series of optimized IP cores, tools, high-level libraries, optimized deep learning models, and example designs. The development stack is high efficient and enables users to fulfill the true potential of AI acceleration on Xilinx hardware platforms. It allows developers to dedicate themselves to fully commit to AI applications rather than the intricacies of the underlying AI and hardware design. The Vitis AI supports multiple deep learning frameworks including Caffe, PyTorch, and TensorFlow. We take the PyTorch in this paper.  Figure 24 shows the flow to deploy the pruned networks on the Xilinx FPGA.

In terms of quantization, first, the input pruned model is preprocessed such as folding batch normalization layers and removing useless nodes. Second, the 32-bit floating-point weights/biases and activation are converted to 8-bit data by the Vitis AI quantizer. The fixed-point model needs less memory bandwidth and then has a higher speed and power efficiency. For the reason that the activation varies with different inputs, the Vitis AI calibrates the activation to improve the accuracy by running several iterations of inference and making a statistic on the activation. After calibration, the deep learning process unit (DPU) deployable model is generated, which can be then compiled. It needs to be noted that there are multiple different DPUs supported for various platforms and the supported DPU for ZCU102 is DPUCZDX8G.

The Vitis AI compiler (VAI_C) compiles the input network to a highly optimized DPU instruction sequence. VAI_C parses the topology of the target model, constructs the internal computation graph as an intermediate representation, performs a series of optimizations, and finally generates the DPU model based on the DPU microarchitecture, which can be deployed on the FPGA. And the compiler that supports DPUCZDX8G is the XCompiler, which stands for the Xilinx intermediate representation (XIR) based compiler.

Table 11 makes a comparison of the changes in the accuracy between the original models and the quantized models. It can be seen that the accuracy decreased by 1.01% and 0.78%, respectively, for the original and pruned VGG13BN, and 1.69% and 2.35%, respectively, for the original and pruned ResNet101. In terms of the accuracy changes, it is obvious that the quantized VGG13BN performs better than the quantized ResNet101. And the accuracy losses are within the range of acceptable.

The Vitis AI Profiler is an all-in-one performance analysis solution for Vitis AI. The profiler can profile both the compiled model running on the DPU and the components implemented by C/C++ or PyThon codes running on a CPU together. With the support of the tool, one can conduct a thorough performance analysis of the AI applications deployed by the Vitis AI, make bottleneck analysis, and do DPU debugging.  Table 12 shows the overall performance of xmodels running based on the DPU. As for the pruned networks, it can be seen that although the model size, the parameters, and FLOPs of VGG13BN are about 3 times, 3 times, and 2 times higher than ResNet101, respectively, the throughput/fps of VGG13BN is about 20 % higher than ResNet101. So parameters and FLOPs are not the only deciding factors of the speed of DNNs, but also the architecture of the network. And the throughput/fps of VGG13BN and ResNet101 for 2-threads mode is about 50% and 80% higher than that of 1-thread mode, respectively. For two threads mode in FPGA, the throughput/fps of the pruned VGG13BN and ResNet101 achieves 151.99 fps and 124.31 fps, respectively, which are 3-4× that of the high-performance Inter(R) Core(TM) i9 10900 CPU.

**Figure 25:**
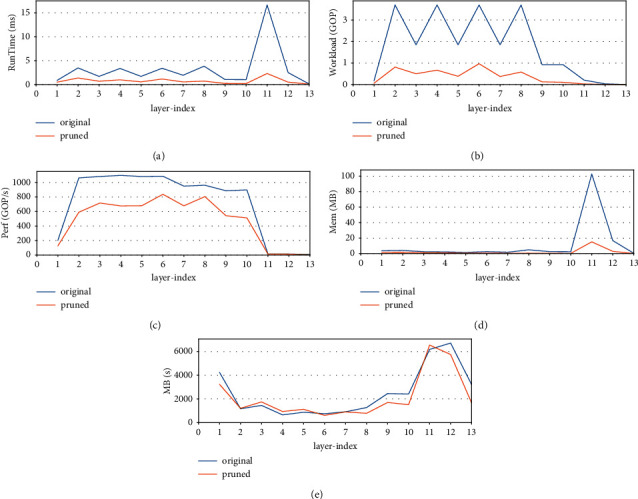
VGG13BN subgraph-wise performance.

**Figure 26:**
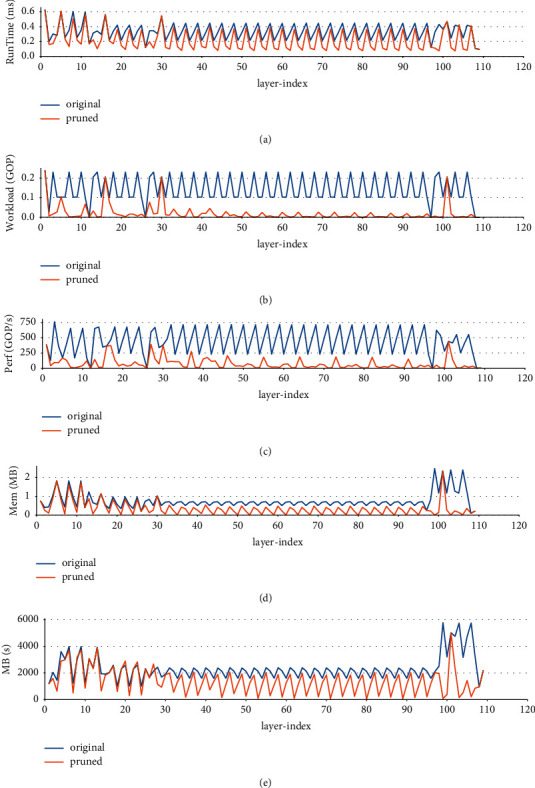
ResNet101 subgraph-wise performance.

The xmodel is a graph object that contains a series of subgraphs corresponding to the operations that can be executed by the DPU. As the networks applied in the paper, the subgraphs also correspond to the convolution operations that fuse the subordinate batch norm and/or the adjacent activation operation. By utilizing the Vitis AI Profiler, the layer-wise, i.e., subgraph-wise, performance is analyzed.  Table 13 shows the DPU layer-wise performance of the original and 2-iteration pruned VGG13BN. The indexes include the workload/GOP (Giga Operations), RunTime/ms, Perf (GOP/s), Mem/MB, and memory access speed G/s. Meanwhile, the table lists the indexes improvement of the pruned network compared with the original model. For clarity, the comparison charts are also represented in Figure 25. Due to the reason that the xmodels of ResNet101 contain more than 100 subgraphs, which is too much to be listed in the table, the paper simply illustrates the layer-wise performance diagrams of the network in Figure 26.

In terms of 2-iteration pruned VGG13BN, it is obvious that the subgraph-wise workload and memory decrease by 79.73% and 70.41%, respectively, on average, and even up to nearly 88% for a specific subgraph. As a result, the overall runtime of the model increases by nearly 4.3×. However, the index subgraph-wise PerfGOP/s decreases by 30.6% on average with a specific subgraph up to 48.24%, and the index G/s also decreases by 8.54% on average with a specific subgraph up to 48.19%. subgraph_11, subgraph_12, and subgraph_13 correspond to the three fully connected (FC) layers. As the data shows, although the three FC layers take the least workload compared with the preceding convolutional layers, they take the most memory and the most run time with the worst performance, especially for the subgraph_11.

As Figure 26 shows, the five indexes of the original ResNet101 have obvious regularity which is consistent with its sequential bottleneck architecture. The three subgraphs with nearly zero workloads and Perf. correspond to the three downsample operations within the network. Bottlenecks found that although the workload and memory decreased obviously compared with the original network, the Perf. also reduces significantly. As a result, although the parameters and FLOPs decreased by almost 90 %, the run time just has a limited increase as Table 12 shows. As for the 3-iteration pruned subgraph-wise characteristics of ResNet101, the workload and memory decrease by 80.56% and 54.13, respectively, on average. Similar to VGG13BN, the index PerfGOP/s decreases by 76.89% on average, and G/s decreases by 37.93% on average.

For the subgraph-layer-wise performance of the pruned networks compared with the original model, the indexes workload/GOP, Runtime/ms, and Mem/MB improve significantly, while the indexes PerfGOP/s and G/s decrease heavily. The underlying principle for the phenomenon and the bottlenecks analyzed such as the three FC layers for VGG13BN provide much room for improvement on both the software level and hardware level. The study provides the research direction for future work.

## 6. Conclusions

This paper provides a “one-shot” solution from software-level to hardware-level acceleration for the applications of DNNs. The high efficient refined pruning method is based on the layer-wise input sparsity of convolutional layers and channel-wise importance indexes of each layer to compress and optimize the DNNs. The pruning is highly efficient in that the parameters and FLOPs can be reduced by more than 86% with less than 1.5% accuracy loss for ResNet50 on CIFAR10, which shows its priority over AutoML for model compression method [49], Prune Train technique [25], and so on. The efficiency of the pruning technique is also verified by extending the experiments with ResNet and VGG13BN to CIFAR100 and ImageNet100 in this paper. And the method can be more refined by adjusting the parameter *η* to achieve a higher pruning ratio of the network with a little loss of accuracy, which just takes more pruning iterations. The refined pruning method can be efficiently extended to all other neural networks with batch normalization operations, which has been adopted by most of the state-of-the-art DNNs. Then, both the original and optimized networks are implemented and accelerated on the Xilinx FPGA platform ZCU102 by utilizing the Vitis AI, and a comprehensive analysis of the subgraph-wise performance concerning the two networks is made. At the same time, the analysis of the xmodels compiled by the Vitis AI and then deployed on the ZCU102 board helps to get a thorough understanding of the neural networks running on FPGA. The analysis results indicate that the model size, the number of parameters, and FLOPs are not the only factors that affect the speed of DNNs; the architecture of the network also plays a key factor in the speed of the model. At the same time, we also found some bottlenecks that restrict the performance of the networks on FPGA. In the future, the research plan is to concentrate on the acceleration of DNNs based on FPGA by designing efficient deep processing units at the hardware level in terms of the characteristics and especially the bottlenecks of the DNN architectures.

## Figures and Tables

**Algorithm 1:**
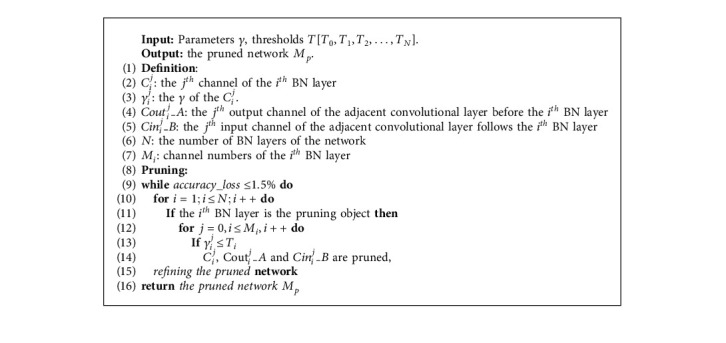
Layer-wise refined pruning.

**Table 1:** ResNet50 on CIFAR10-1.

Pruning iteration	Params (M)	FLOPS (M)	Pruned ratio (%)		Val. acc. (%)
			Params	FLOPS	
Baseline	23.52	1305.83	—	—	93.6
1	15.16	825.06	35.54	36.82	94.37
2	9.97	528.43	57.61	59.53	94.56
3	6.88	361.83	70.75	72.29	94.45
4	5.20	264.20	77.89	79.77	94.53
5	4.31	211.08	81.68	83.84	94.46
6	3.77	178.84	83.97	86.30	93.98
7	3.44	158.99	85.37	87.82	93.27
8	**3.25**	**148.32**	**86.18**	**88.64**	**92.37**
9	3.11	142.16	86.78	89.11	92.09

The bold values mean that the best optimized network is obtained at the 8th iteration.

**Table 2:** ResNet101 on CIFAR100-1.

Pruning iteration	Params (M)	FLOPS (M)	Pruned ratio (%)		Val. acc. (%)
			Params	FLOPS	
Baseline	42.71	2523.36	—	——	71.94
1	24.47	1457.96	42.69	42.22	72.75
2	14.64	870.15	65.71	65.52	72.45
3	9.69	561.83	77.31	77.73	72.82
4	6.95	386.05	83.72	84.70	72.14
5	**5.5**	**286.38**	**87.12**	**88.65**	**71.65**
6	4.48	225.57	89.51	91.06	70.72

The bold values mean that the best optimized network is obtained at the 5th iteration.

**Table 3:** ResNet50 on CIFAR10-2.

Pruning iteration	Params (M)	FLOPS (M)	Pruned ratio (%)		Val. acc. (%)
			Params	FLOPS	
Baseline	23.52	1305.83	—	—	93.6%
1	9.61	486.31	59.14	62.76	94.24%
2	5.06	245.71	78.49	81.18	93.53%
3	**3.70**	**178.35**	**84.27**	**86.34**	**92.78**%
4	3.28	155.4	86.05	88.10	92.14%

The bold values mean that the best optimized network is obtained at the 3rd iteration.

**Table 4:** ResNet101 on CIFAR100-2.

Pruning iteration	Params (M)	FLOPS (M)	Pruned ratio (%)		Val. acc. (%)
			Params	FLOPS	
Baseline	42.71	2523.36	—	—	71.94
1	14.72	901.45	65.53	64.28	72.53
2	7.77	464.26	81.80	81.60	72.43
3	**5.36**	**298.31**	**87.45**	**88.19**	**71.21**
4	4.30	222.70	89.93	91.17	69.38

The bold values mean that the best optimized network is obtained at the 3rd iteration.

**Table 5:** ResNet50 on CIFAR10.

Method	Pruning ratio (%)		Val. acc. (%)
	Para.	FLOPs	
AutoML for model compression (93.53%) [49]	60	**—**	+0.12
Prune Train (94.2%) [25]	—	70	−1.10
Automatic neural network compression (93.55%) [26]	50	—	−1.51
Pruning it Yourself (92.85%) [50]	41.52	34.18	−0.16
MS-KD 0.6 (95.36%) [51]	84.01	84.10	−0.24
Ours scheme 1 Iteration-6 (93.6%)	83.97	86.30	+0.40

**Table 6:** ResNet50 on ImageNet100.

Pruning iteration	Params (M)	FLOPS (M)	Pruned ratio (%)		Val. acc. (%)
			Params	FLOPS	
Baseline	23.71	4116.69	—	—	79.98
1	13.33	2388.97	43.78	41.97	80.35
2	8.64	1569.34	63.56	61.88	80.39
3	6.32	1156.42	73.34	71.91	79.82
4	**5.10**	**895.66**	**78.49**	**78.24**	**79.30**
5	4.30	739.39	81.86	82.04	78.08

The bold values mean that the best optimized network is obtained at the 4th iteration.

**Table 7:** ResNet101 on ImageNet100.

Pruning iteration	Params (M)	FLOPS (M)	Pruned ratio (%)		Val. acc. (%)
			Params	FLOPS	
Baseline	42.71	7842.56	—	—	80.05
1	13.64	2589.43	68.06	66.98	80.61
2	9.08	1708.27	78.74	78.22	80.24
3	**6.74**	**1226.4**	**84.22**	**84.36**	**79.49**
4	5.43	963.8	87.29	87.71	78.68

The bold values mean that the best optimized network is obtained at the 3rd iteration.

**Table 8:** VGG13BN on CIFAR10.

Pruning iteration	Params (M)	FLOPS (M)	Pruned ratio (%)		Val. acc. (%)
			Params	FLOPS	
Baseline	28.35	248.33	—	—	90.72
1	12.86	183.29	54.64	26.19	91.52
2	6.55	104.01	76.90	58.12	91.64
3	**3.67**	**60.15**	**87.05**	**75.78**	**90.05**
4	1.98	37.23	93.02	85.01	87.81

The bold values mean that the best optimized network is obtained at the 3rd iteration.

**Table 9:** VGG13BN on CIFAR100.

Pruning iteration	Params (M)	FLOPS (M)	Pruned ratio (%)		Acc. (%)
			Params	FLOPS	
Baseline	28.72	248.70	—	—	65.37
1	**11.40**	**164.72**	**60.31**	**33.77**	**66.2**
2	6.19	92.96	78.45	62.62	64.36

The bold values represent that the best optimized network is obtained at the 1st iteration.

**Table 10:** VGG13BN on ImageNet100.

Pruning iteration	Params (M)	FLOPS (G)	Pruned ratio (%)		Acc. (%)
			Params	FLOPS	
Baseline	129.39	11.34	—	—	78.85
1	49.67	5.04	61.61	55.53	79.00
2	**19.71**	**2.33**	**84.76**	**79.47**	**77.85**
3	8.57	1.11	93.37	90.23	74.65

The bold values mean that the best optimized network is obtained at the 2nd iteration.

**Table 11:** Accuracy comparison after quantization.

ImageNet100	Original model (acc.)		Pruned model (acc.)	
	FP32	INT8	FP32	INT8
VGG13BN	78.85	78.05	77.85	77.23
ResNet101	79.08	77.74	78.28	76.44

**Table 12:** Overall performance of xmodel on FPGA.

Compiled network	Size (MB)	Throughput (fps)	
		1-thread	2-threads
Original VGG13BN	130.03	23.14	35.14
Pruned VGG13BN	20.05	87.89	151.99
Original ResNet101	45.40	36.90	68.30
Pruned ResNet101	7.54	67.72	124.31

**Table 13:** xmodel layer-wise performance of VGG13BN.

Sub	Workload (GOP)			Run time (ms)			PerfGOP (s)			Mem (MB)			Mem-access *G*(s)		
	Orig.	Prun.	Imp. (%)	Orig.	Prun.	Imp. (%)	Orig.	Prun.	Imp. (%)	Orig.	Prun.	Imp. (%)	Orig.	Prun.	Imp. (%)
S1	0.17	0.06	64.16	0.86	0.50	42.48	200.70	125.39	−37.52	3.67	1.61	56.18	4.24	3.23	−23.80
S2	3.70	0.81	78.07	3.48	1.38	60.48	1064.27	590.62	−44.50	4.08	1.66	59.26	1.17	1.21	3.06
S3	1.85	0.50	72.86	1.71	0.70	58.99	1083.59	717.05	−33.83	2.48	1.22	50.68	1.45	1.75	20.27
S4	3.70	0.67	81.90	3.37	0.99	70.64	1098.54	677.35	−38.34	2.21	0.93	57.96	0.66	0.94	43.27
S5	1.85	0.39	79.19	1.71	0.57	66.92	1082.96	680.57	−37.16	1.50	0.64	57.64	0.88	1.12	28.09
S6	3.70	0.97	73.81	3.40	1.16	66.01	1087.01	837.61	−22.94	2.57	0.71	72.27	0.75	0.62	−18.43
S7	1.85	0.38	79.73	1.95	0.55	71.58	950.51	678.63	−28.60	1.78	0.50	71.89	0.92	0.91	−1.00
S8	3.70	0.58	84.27	3.84	0.72	81.21	962.73	805.68	−16.31	4.90	0.57	88.36	1.27	0.79	−38.05
S9	0.93	0.13	86.27	1.04	0.24	77.47	886.72	542.47	−38.82	2.56	0.40	84.38	2.45	1.70	−30.66
S10	0.93	0.11	88.65	1.03	0.21	80.10	898.00	511.74	−43.01	2.49	0.31	87.48	2.41	1.52	−37.17
S11	0.21	0.03	85.44	16.65	2.32	86.09	12.35	13.08	5.94	102.79	15.16	85.25	6.18	6.55	5.99
S12	0.03	0.01	82.35	2.50	0.51	79.65	13.42	11.48	−14.45	16.79	2.93	82.57	6.71	5.75	−14.37
S13	0.00	0.00	—	0.13	0.10	25.58	6.35	3.29	−48.24	0.41	0.16	61.35	3.21	1.66	−48.19

Orgi.: the original network; Prun.: the pruned network; Imp.: the improvement.

## Data Availability

The data used to support the findings of this study are included within the article. And the datasets used in the study are public datasets which can be accessed easily online.
